# C-Terminus of the B-Chain of Relaxin-3 Is Important for Receptor Activity

**DOI:** 10.1371/journal.pone.0082567

**Published:** 2013-12-11

**Authors:** Fazel Shabanpoor, Ross A. D. Bathgate, John D. Wade, Mohammed Akhter Hossain

**Affiliations:** 1 Florey Institute for Neuroscience & Mental Health, University of Melbourne, Melbourne, Victoria, Australia; 2 School of Chemistry, University of Melbourne, Melbourne, Victoria, Australia; 3 Florey Department of Neuroscience and Mental Health, University of Melbourne, Melbourne, Victoria, Australia; 4 Department of Biochemistry and Molecular Biology, University of Melbourne, Melbourne, Victoria, Australia; Medical School of Hannover, Germany

## Abstract

Human relaxin-3 is a neuropeptide that is structurally similar to human insulin with two chains (A and B) connected by three disulfide bonds. It is expressed primarily in the brain and has modulatory roles in stress and anxiety, feeding and metabolism, and arousal and behavioural activation. Structure-activity relationship studies have shown that relaxin-3 interacts with its cognate receptor RXFP3 primarily through its B-chain and that its A-chain does not have any functional role. In this study, we have investigated the effect of modification of the B-chain C-terminus on the binding and activity of the peptide. We have chemically synthesised and characterized H3 relaxin as C-termini acid (both A and B chains having free C-termini; native form) and amide forms (both chains’ C-termini were amidated). We have confirmed that the acid form of the peptide is more potent than its amide form at both RXFP3 and RXFP4 receptors. We further investigated the effects of amidation at the C-terminus of individual chains. We report here for the first time that amidation at the C-terminus of the B-chain of H3 relaxin leads to significant drop in the binding and activity of the peptide at RXFP3/RXFP4 receptors. However, modification of the A-chain C-terminus does not have any effect on the activity. We have confirmed using circular dichroism spectroscopy that there is no secondary structural change between the acid and amide form of the peptide, and it is likely that it is the local C-terminal carboxyl group orientation that is crucial for interacting with the receptors.

## Introduction

Relaxin-3 is the most recently discovered member of insulin/relaxin superfamily of polypeptide hormones [1]. Using phylogenetic analysis, relaxin-3 has been postulated to be the likely ancestor of the relaxin subfamily due to the high degree of homology of its orthologs in various species from fish to mammals [2]. Like the rest of peptides in this family, relaxin-3 is expressed as a single-chain prohormone that is converted to the mature peptide by formation of three disulfide bonds (one intra-chain and two inter-chains) followed by excision of the C-chain which connects the A-chain to the B-chain [3]. Its tertiary structure has been determined using solution NMR spectroscopy which shows a high degree of similarity to known structures from other members of the family particularly in the regions held together by the three disulphide bonds [4].

Human relaxin-3 (H3 relaxin) binds and activates its cognate receptor known as relaxin-family peptide receptor-3 (RXFP3) [5], [6]. It also interacts with RXFP4, the native receptor for insulin-like peptide 5 (INSL5), as well as RXFP1, the native receptor for human relaxin-2 (H2 relaxin) [7] with high affinity. It is a neuropeptide that is primarily expressed in the brain with the highest level detected in the nucleus incertus (NI) of the mouse [1], [8], rat [6], macaque [9] and human [6]. Neurons from the NI project to many regions of the brain where its distribution overlaps with that of RXFP3 [10], [11]. The conserved tissue distributions of relaxin-3 and RXFP3 together with behavioral data from animal studies suggest that relaxin-3 has roles in stress/anxiety [12], cognition [13] and appetite regulation [14], [15].

Structure-activity relationship studies have shown that relaxin-3 interacts with its receptor primarily through its B-chain [16]-[18]. In a more recent study, we developed an analogue of H3 relaxin which selectively binds to and activates RXFP3 with no cross-activity at RXFP1 [19]. A C-terminally truncated single B-chain antagonist has also been developed which binds RXFP3 with full affinity and has been demonstrated to antagonise food intake in rats [20]. These selective agonist and antagonist peptides have since been used as powerful tools for the in vivo study of the physiological roles of H3 relaxin. However, while these peptide modulators are very useful, they have to be administered via the i.c.v. route. Therefore, small molecule drugs are of great interest for targeting RXFP3.

**Figure 1:**
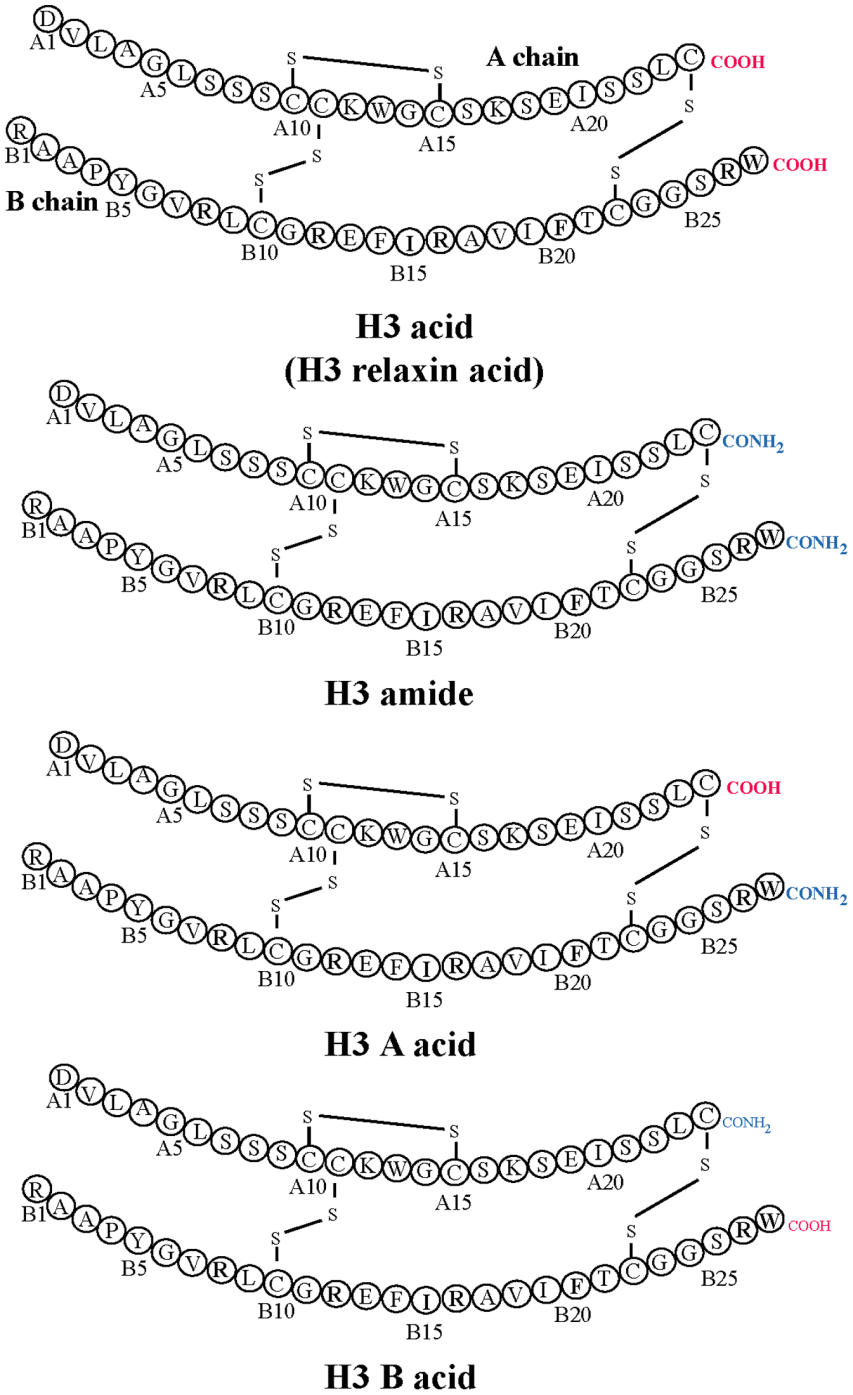
Primary structures of H3 peptides: H3 acid, H3 amide, H3 B acid, H3 A acid. The C-terminal differences of A- and B-chains are highlighted.

In this endeavour, a small molecule, 135PAM1, has recently been discovered by Alvarez-Jaimes et al. [21] They reported that it was an allosteric modulator of RXFP3 that worked in the presence of an orthosteric agonist, H3 relaxin. However, the state of the H3 relaxin’s C-terminus was found to be crucial for the allosteric activity of 135PAM1. 135PAM1 was shown to be active only when used with the non-native amidated form of H3 relaxin (H3 amide; Figure 1). Since 135PAM1 was unable to act in the presence of the native form (acid) of H3 relaxin (H3 acid; Figure 1), it has very limited utility as a drug. Interestingly, our initial studies suggested that the amidated and free-acid forms of H3 relaxin [4] were equipotent at RXFP3. However, the study conducted by Alvarez-Jaimes et al. [21] contradicts this finding and demonstrated that H3 acid was more potent than the chemically synthesised amidated form.

In order to investigate this difference in the activity of the C-terminal acid and amide form of H3 relaxin, we have chemically prepared four different H3 relaxin variants (Figure 1). Each was fully characterised for its binding and activity at both RXFP3 and RXFP4, and analysed its secondary conformation using circular dichroism study. We report here for the first time that B-chain of H3 relaxin needs to be a C-terminal acid to high biological activity.

## Materials and Methods

### Materials

All 9-fluroenylmethoxycarbonyl (Fmoc) protected L-α-amino acids and 2-(1H-benzotriazol-1-yl)-1,1,3,3-tetramethyluronium hexafluorophosphate (HBTU) were obtained from GL Biochem (Shanghai, China). Fmoc-Cys(Acm)-Ac-S-TentaGel (0.22 mmol/g) resin was obtained from Rapp Polymere GmbH (Tübingen, Germany). Fmoc-Trp(Boc)-PEG-PS (0.23 mmol/g) and Fmoc-PAL-PEG-PS (0.19 mmol/g) resins were purchased from Applied Biosystems (Melbourne, Australia). All peptide synthesis solvents were obtained from MERCK (Kilsyth, VIC, Australia). 2, 2'-dipyridyl disulfide (DPDS) was from Fluka (Bucha, Switzerland) and TFMSA from MP Biomedicals (Seven Hills, NSW, Australia). All other reagents for peptide synthesis and bovine serum albumin (BSA) and 4-(2-hydroxyethyl)-1-piperazineethanesulfonic acid (HEPES) were obtained from Sigma-Aldrich (Sydney, NSW). Chlorophenolred-β-D-galactopyranoside (CPRG) was obtained from Roche (Indianapolis, USA). All other reagents for cells culture were sourced from Trace Biosciences (Castle Hill, NSW).

### Peptide synthesis

Linear peptides with a C-terminal amide were assembled on PAL-PEG-PS resin and the A- and B-chains with C-terminal acid were synthesised using Fmoc-Cys(Acm)-Ac-S-TentaGel and Fmoc-Trp(Boc)-PEG-PS respectively. The peptides were synthesised using a CEM Liberty™ microwave peptide synthesizer (AI Scientific, Scarborough, QLD, Australia). All peptides were synthesised using a 0.1 mmol scale with excess of Fmoc-protected amino acids, HBTU and DIPEA (5∶5∶10). N^α^ deprotection was carried out using 20% piperidine in DMF. The peptides were cleaved from solid support using a cocktail of TFA:DoDt:H_2_O:TIPS (94∶2.5∶2.5∶1, v/v) for 2 hr. The combination of the two chains was carried out using sequential regioselective disulphide bond formation as previously described [22]-[24]. The crude peptides were analyzed and purified by RP-HPLC on Waters XBridge™ columns (4.6×250 mm, C18, 5 µm; 19×150 mm, C18, 5 µm) using H_2_O and acetonitrile with 0.1% TFA as solvent A and B respectively. The characterization of peptides was carried using MALDI-TOF/TOF mass spectroscopy (Bruker Daltonics, Germany) with sinapinic acid as matrix.

### Circular dichroism (CD) spectroscopy

The secondary structural changes of the peptides was measured by recording their CD spectra on JASCO model J815 spectropolarimeter as previously described [25]. The CD spectra were recorded in phosphate buffer saline (10 mM) with peptide concentrations made up to 0.2 µg/µl. Helix content of peptide is directly proportional to mean residue ellipticity at 222 nm [θ]_222_. The [θ]_222_ value for each peptide was determined from the CD spectra that were measured at 25 °C. One hundred percent helicity was calculated by using the formula ^max^[θ]_222_  =  − 40000 × [(1−2.5/n)] + (100 + T), where n =  number of amino acid residues and T =  temperature of the peptide solution in °C [26]. Percentage helicity was then calculated as 100 × [θ]_222_/^max^[θ]_222_.

### Receptor binding assay

The receptor binding affinity of the peptides was measured using CHO-K1 cells stably expressing RXFP3 and RXFP4. The competition binding assays were carried out as previously described [24], [27]. Briefly, a single concentration of Eu-labelled I5/R3 (0.5 nM) or Eu-labelled mouse INSL5 (2.5 nM) were used in presence of increasing concentration of peptides. The data were analyzed using GraphPad PRISM 5 (GraphPad Inc., San Diego, USA). The europium binding curves were fitted to a one site competition curve and the pKi values were expressed as the mean ± SEM of at least three independent experiments with triplicate determinations within each assay.

### cAMP activity assay

The ability of the peptides to activate the receptors RXFP3/RXFP4 was assessed by measuring their influence on cAMP signalling in CHO-K1 cells stably expressing RXFP3 or RXFP4 transfected with a pCRE-β-galactosidase reporter gene construct as previously described [23]. A single concentration of forskolin was used to stimulate cAMP signalling in cells expressing RXFP3 (1 µM) and RXFP4 (5 µM) and peptide induced inhibition of cAMP signalling was measured by adding increasing concentrations of the peptides (0.01 nM – 1 µM). The ability of the peptides to inhibit forskolin-induced cAMP response (pEC50) was measured and expressed as mean ± SEM from three independent experiments with triplicate determinations within each assay. The inhibition curves were fitted to a single-site sigmoidal dose-response curve with variable slope using Graphpad PRISM 5.

## Results, Discussion, and Conclusion

To study the effects of amidation at the C-termini of H3 relaxin, we chemically synthesized four analogues: H3 acid (both A and B chains C-termini acid), H3 amide (both A and B chains C-termini amide), H3 B acid (A chain C-terminus amide, B chain C-terminus acid) and H3 A acid (A chain C-terminus acid, B chain C-terminus amide) (Figure 1). A regioselective disulfide bond formation approach was used to complete the synthesis of all H3 relaxin analogues. Three orthogonal cysteine S-protecting groups (Trt, tBu and Acm) were used that allowed the sequential formation of three disulfide bonds. Solid phase synthesis of the separate, selectively S-protected A- and B-chains followed by their purification and subsequent stepwise formation of each of the three disulphide bonds via oxidation, thioloysis, and iodolysis [28]-[30] led to the successful preparation of H3 peptides. Their analytical RP-HPLC profiles and MALDI TOF MS spectrometric analysis (H3 acid theoretical, MH+5501.52 found, MH+5500.63; H3 amide theoretical, MH+5499.52; found, MH+5499.34; H3 A acid theoretical, MH+5500.52; found, MH+5501.6; H3 B acid theoretical, MH+5500.52; found, MH+5501.2) revealed highly purified synthetic products. The peptide content and amino acid composition were determined by amino acid analysis (H3 acid 69.78%; H3 amide 65.06; H3 A acid 77.87%; H3 B acid 58.1%). The synthesis of amidated peptides was found to be easier and the yield was relatively higher (10-12%) compared to the free acid forms (6%).

**Figure 2:**
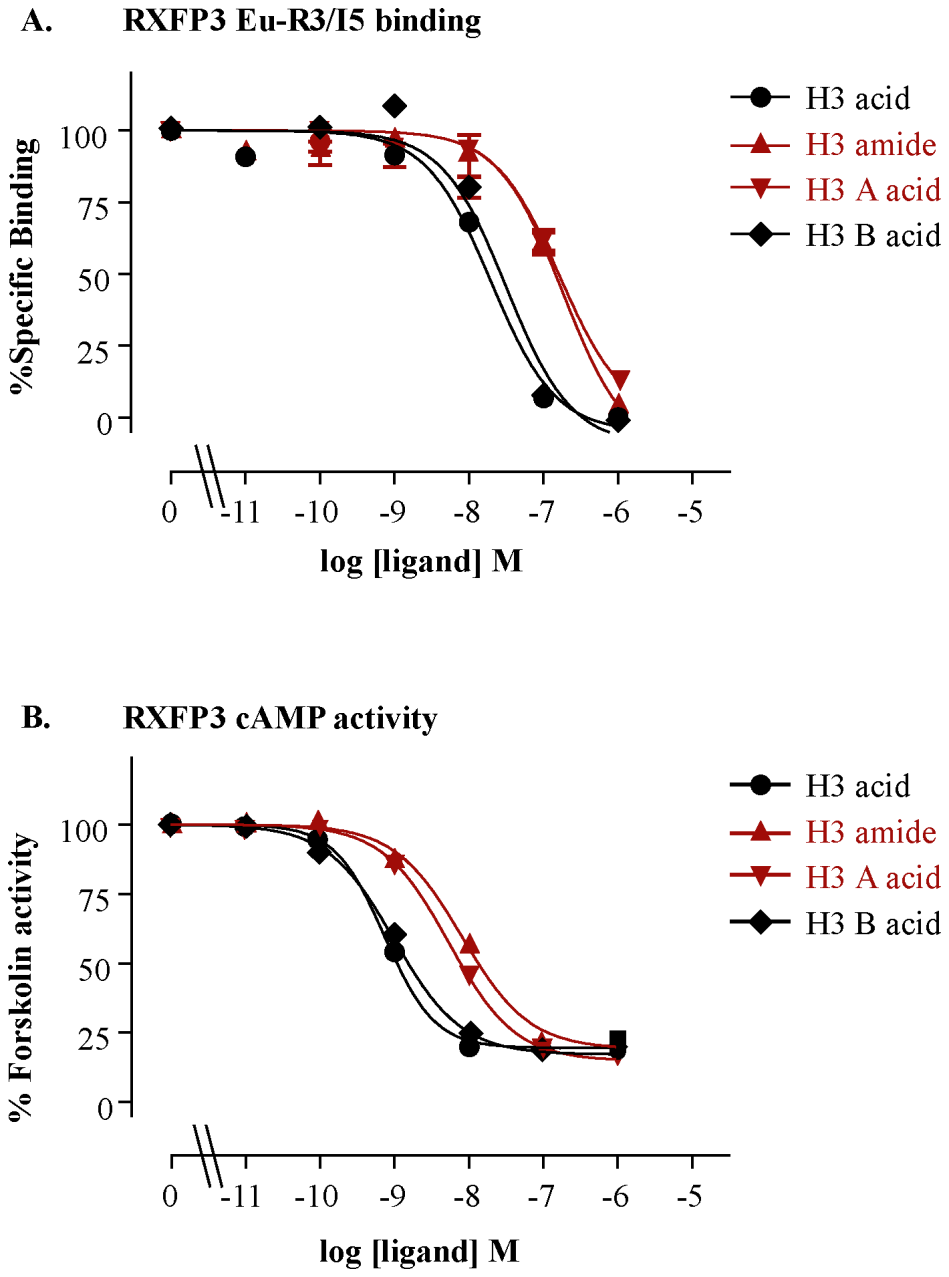
Receptor binding affinity (pK_i_) and cAMP activity (pEC­50) curves of H3 relaxin and its variants at RXFP3. **A)** Competition binding curves of H3 relaxin variants using a single concentration of Eu-I5/R3 (0.5 nM) in the presence of increasing concentrations of the H3 relaxin peptides. **B)** RXFP3 activation curves of H3 relaxin variants measured in a cAMP activity assay in the presence of 5 µM forskolin. The data were expressed as mean ± SEM. from at least three independent experiments.

The peptides were tested for their ability to bind to and activate their native receptor, RXFP3. The H3 acid showed about 10 times higher affinity and potency compared with H3 amide at RXFP3 (Figure 2, Table 1). This result is consistent with the results published by Alvarez-Jaimes et al. [21].

**Table 1:** Pooled binding affinity (pKi) and cAMP activity (pEC50) data for H3 acid variants at RXFP3 and RXFP4.

Ligand	RXFP3		RXFP4	
	Eu-H3/I5 pKi	cAMP pEC50	Eu-INSL5 pKi	cAMP pEC50
H3 acid	7.73±0.04 (3)	9.17±0.02 (3)	8.06±0.03 (3)	8.94±0.13 (3)
H3 amide	6.80±0.11 (4)**	8.14±0.09 (3)**	7.40±0.12 (3)*	8.38±0.06 (3)*
H3 A-acid	6.82±0.11 (3)**	8.31±0.11 (3)**	6.93±0.02 (3)**	7.46±0.11 (3)**
H3 B-acid	7.62±0.07 (3)	9.02±0.05 (3)	7.72±0.10 (3)	8.88±0.27 (3)

**Figure 3:**
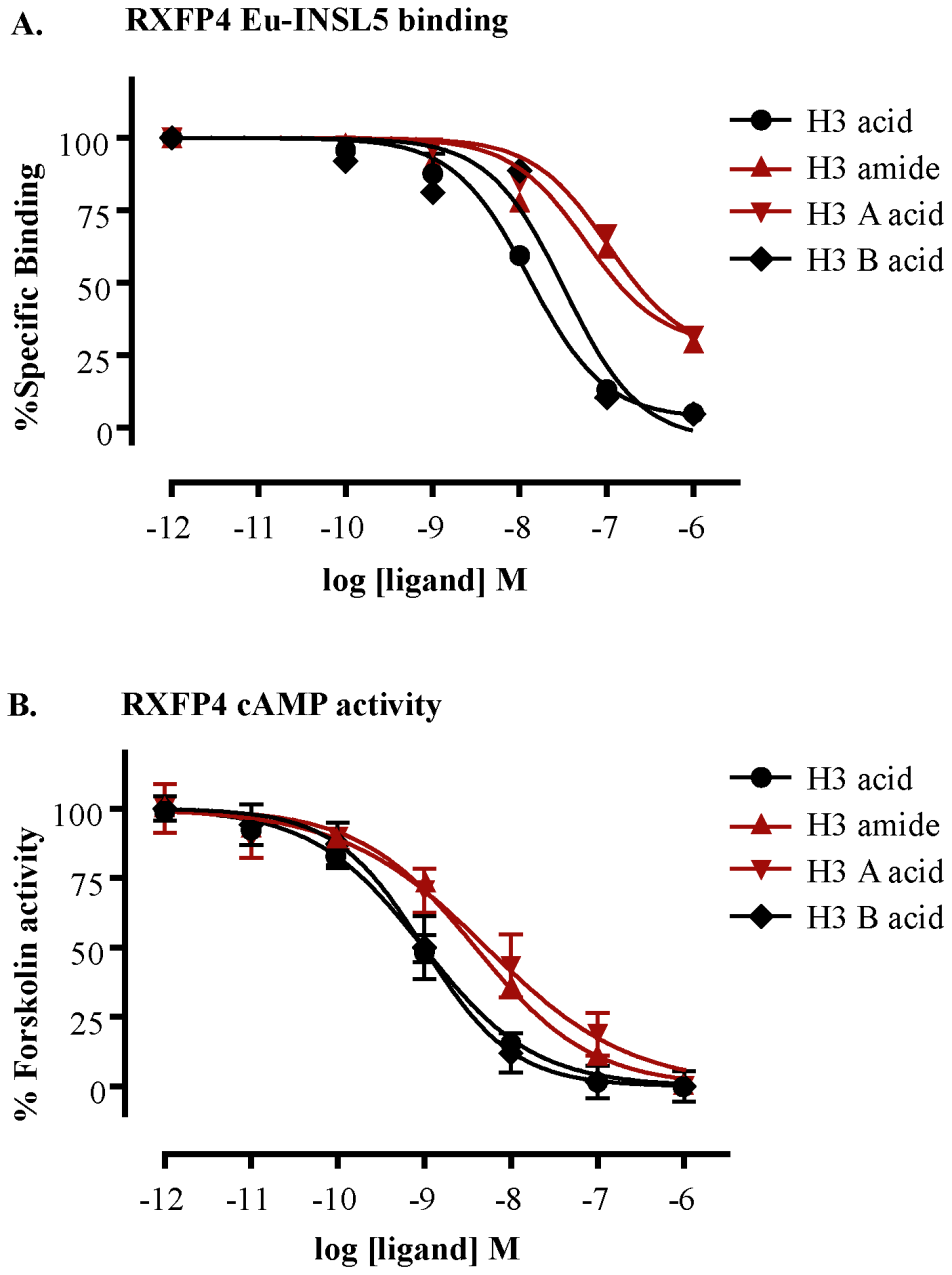
Receptor binding affinity (pK_i_) and cAMP activity (pEC­50) curves of H3 relaxin and its variants at RXFP4. **A)** Competition binding curves of H3 relaxin variants at RXFP4 using a single concentration of Eu-INSL5 (2.5 nM) in the presence of increasing concentrations of the H3 relaxin peptides. **B)** RXFP4 activation curves of H3 relaxin variants measured in a cAMP activity assays in the presence of 1 µM forskolin. The data were expressed as mean ± SEM from at least three independent experiments.

**p<0.01; vs H3 relaxin acid. p<0.05;

Relaxin-3 is closely related to insulin-like peptide 5 (INSL5) as they both belong to the same branch in the phylogenetic tree of the relaxin family. In addition, the native receptors of relaxin-3 and INSL5, RXFP3 and RXFP4 respectively, belong to the same class of G-protein coupled receptors. On the basis of such close relationship between ligands (relaxin-3 and INSL5) and receptors (RXFP3 and RXFP4), it is most likely that relaxin-3 interacts with RXFP3 in a similar way to INSL5 interaction with RXFP4. Therefore, we tested all four H3 relaxin analogues on RXFP4. As expected, the H3 acid peptide showed higher affinity and potency (6-7 times) at RXFP4 as well (Figure 3, Table 1). This result is consistent with our recent observation for INSL5 where we showed that the C-termini of INSL5 need to be an acid for full RXFP4 activity [31].

However, to determine specifically whether the C-terminus of the both chains (A and B) or either of the chain (A or B) is important for RXFP3/RXFP4 activity, we tested the activity of two other analogues, H3 A acid and H3 B acid. Interestingly, the H3 B acid was found to be almost equipotent to H3 acid, and H3 A acid was found to be almost equipotent to H3 amide on both RXFP3 (Figure 2, Table 1) and RXFP4 receptors (Figure 3, Table 1). This result clearly demonstrates that it is the C-terminus of the B-chain which is critical for the high affinity and potency of H3 relaxin at both RXFP3 and RXFP4 receptors. The nature of the C-terminus (acid or amide) of the A-chain does not affect the activity of H3 relaxin.

The bioactivity of many polypeptide hormones often requires a form of post-translational modification of their precursors. An example of a widespread modification is C-terminal amidation which often leads to an increase in bioactivity of peptides [32]–[35]. In some cases, amidation of the C-terminus has been reported to have detrimental effect on the activity of peptides [31], [36]. Oxytocin, for example, is only active as a C-terminal amide form whereas angiotensin II (AII) is active as a C-terminal acid form [37]. In the case of relaxin family peptides, H2 relaxin and INSL3, both peptides are equally active either in acid or amide form [16], [38], [39]. Given that it is technically easier and often faster to synthesize peptide amides which only need simple acylation of the C-terminal amino acid onto a support with a suitably functionalized linker. In contrast, a more expensive resin pre-loaded with the first amino acid attached to an appropriate linker can be purchased or assembled using more involved ester bond formation chemistry. This convenience of preparation of peptide amides has led us to prepare most of our in-house peptides in this form. However, in the case of INSL5, we recently amidated the C-termini of both of its A and B chains and showed that C-termini were required to be free (acid) for high RXFP4 activity. But we are yet to investigate the effects of individual chains of INSL5. Within the insulin-relaxin peptide family, this is the first study to investigate the role of the C-terminus of the individual chains and to show that the B-chain C-terminus, not the A-chain, of H3 relaxin needs to be in the carboxylic acid form for high RXFP3/RXFP4 affinity and potency.

**Figure 4:**
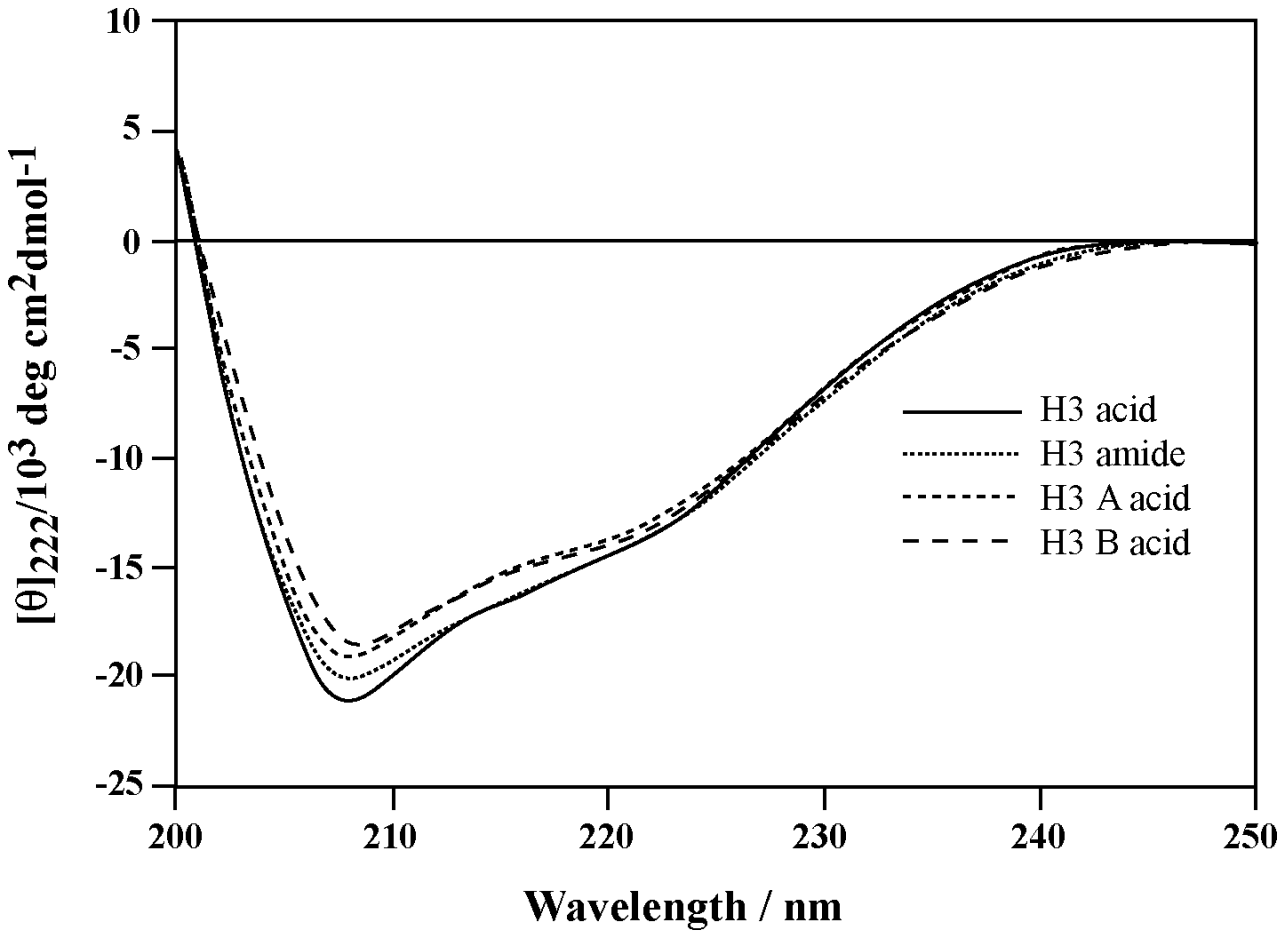
CD spectra of human H3 relaxin variants measured at a concentration of 0.2 µg/µl in PBS (pH 7.4) at room temperature. The four peptides showed similar spectra with calculated helicity ranging from 34–38%.

The question arises now whether the peptide’s structure is altered due to amidation, itself a subtle change at the C-terminus of the B-chain, and if such alteration caused a significant drop in the binding and activation of both RXFP3 and RXFP4. We thus analysed the secondary structure of all four peptides. The level of α-helicity was determined by CD spectroscopy. The CD spectra showed a typical α-helical pattern with double minima at 208 and 222 nm (Figure 4). The mean residual ellipticity (MRE) at 222 nm, [θ]_222,_ was used to calculate the helix content [26]. The [θ]_222_ values for H3 acid, H3 amide, H3 A-acid and H3 B-acid were found to be –13532.5, –13694.4, –13134.6 and –12438.2 which correspond to an α-helix content of 37.59%, 38.04%, 36.48%, and 34.55% respectively. Clearly, there was no observable perturbation in the secondary structure of any of these peptides. Therefore the loss of activity of amidated B-chain analogues is not likely due to loss of structural integrity of the peptides.

**Figure 5:**
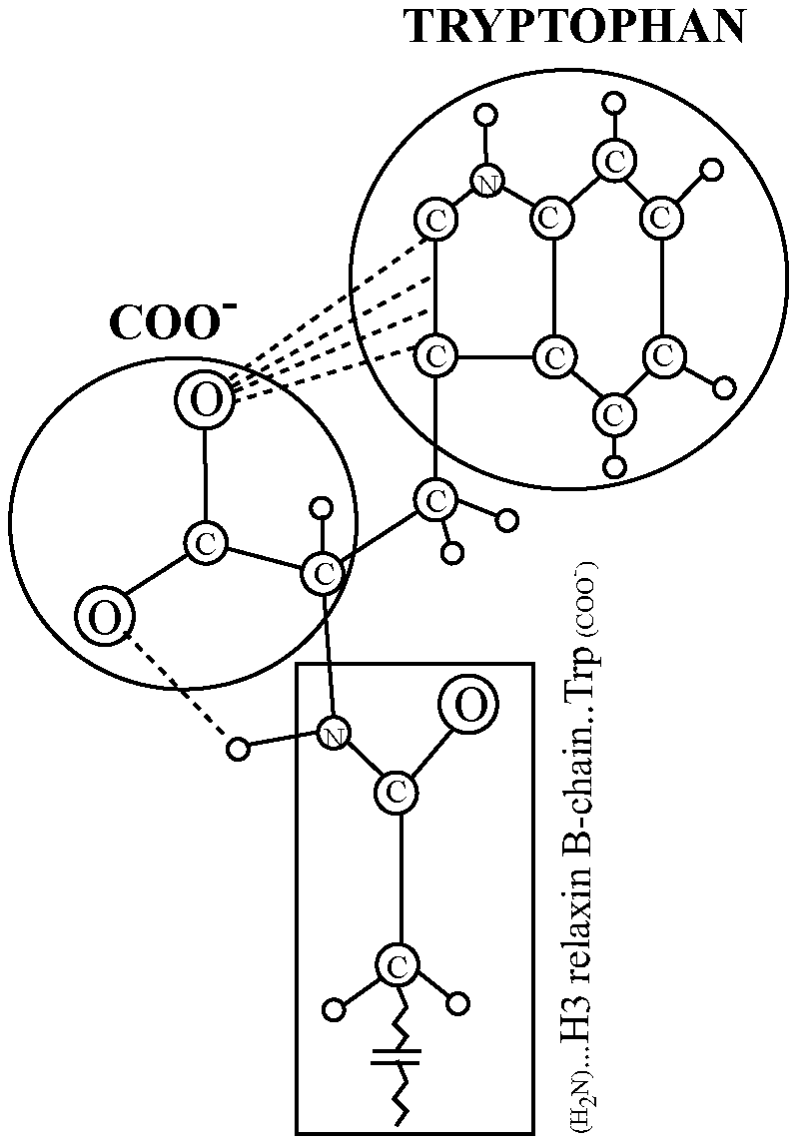
Proposed mechanism of interaction between C-terminal carboxyl-group and aromatic ring of tryptophan side-chain. The specific interaction of the oxygen with the pyrrole ring, and the H-bond between the carboxyl oxygen and the α -NH amide of Trp, as shown in the figure, is speculative.

It is possible that proper orientation of the C-terminus carboxyl oxygen is crucial for high RXFP3/RXFP4 affinity and potency and that this is maintained by the interaction of the carboxyl oxygen with the electrons of the whole aromatic system of the indole moiety of Trp residue. This hypothesis is supported by the fact that the interaction of an aromatic ring (Phe residue) with oxygen of the carbonyl group has been demonstrated from examination of 170 Phe residues in 28 protein crystal structures and also by *ab initio* calculations [40]. It was shown that the influence of the aromatic side chain on the local conformation of the AII peptide (agonist) was very specific and such local conformation was very critical for full biological activity [37]. However, when Phe residue in AII was replaced with a non-aromatic cyclohexyl alanine, the peptide became an antagonist indicating the importance of the interaction between the aromatic ring and the free –COOH group [37]. Based on the NMR structure of H3 relaxin [4] and AII [37], we propose a mechanism of interaction between aromatic side chain of Trp and COOH group in the B-chain of H3 relaxin (Figure 5).

The requirement for a free B chain C-terminal carboxylate may also reflect the need for the presence of a positive charge in the receptors which is involved in an electrostatic interaction with the acid form. We have recently demonstrated that Arg residues in the central alpha-helix of the B-chain are involved in electrostatic interactions with Glu and Asp residues in the RXFP3 receptor exoloops [41]. Molecular modelling of the interaction of H3 relaxin with RXFP3 suggested that the C-terminal Trp residue may be interacting with residues in the RXFP3 TM domains. However the precise residue(s) with which it is interacting are not yet known. The current study suggests that the Trp may be interacting with a residue with a positive charge.

In conclusion, we have chemically prepared four complex two chain, three disulfide-bonded H3 relaxin peptides bearing either or both of C-terminal acids or amides. We found that C-terminus of the B-chain of H3 relaxin is crucial for both binding and activation of RXFP3 and RXFP4 receptors. Therefore any modification to the peptide termini of H3 relaxin will need to be taken into careful consideration as it may lead to undesirable effects on the activity of the peptides. Given the difficulty of the synthesis and poor recovery of native H3 acid peptide, H3 B acid, which is easier to synthesize and as potent as native peptide, would be a valuable template to further study the neurological function of H3 and RXFP3. On the other hand, H3 A acid, which is as potent as H3 amide, might be an alternative template for the study of the allosteric activity of the 135PAM1 small compound.
